# Large-Scale, High-Resolution Multielectrode-Array Recording Depicts Functional Network Differences of Cortical and Hippocampal Cultures

**DOI:** 10.1371/journal.pone.0105324

**Published:** 2014-08-15

**Authors:** Shinya Ito, Fang-Chin Yeh, Emma Hiolski, Przemyslaw Rydygier, Deborah E. Gunning, Pawel Hottowy, Nicholas Timme, Alan M. Litke, John M. Beggs

**Affiliations:** 1 Santa Cruz Institute for Particle Physics, University of California Santa Cruz, Santa Cruz, California, United States of America; 2 Department of Physics, Indiana University, Bloomington, Indiana, United States of America; 3 Microbiology and Environmental Toxicology Department, University of California Santa Cruz, Santa Cruz, California, United States of America; 4 Faculty of Physics and Applied Computer Science, AGH University of Science and Technology, Kraków, Poland; 5 Institute of Photonics, University of Strathclyde, Glasgow, United Kingdom; University of Genova, Italy

## Abstract

Understanding the detailed circuitry of functioning neuronal networks is one of the major goals of neuroscience. Recent improvements in neuronal recording techniques have made it possible to record the spiking activity from hundreds of neurons simultaneously with sub-millisecond temporal resolution. Here we used a 512-channel multielectrode array system to record the activity from hundreds of neurons in organotypic cultures of cortico-hippocampal brain slices from mice. To probe the network structure, we employed a wavelet transform of the cross-correlogram to categorize the functional connectivity in different frequency ranges. With this method we directly compare, for the first time, in any preparation, the neuronal network structures of cortex and hippocampus, on the scale of hundreds of neurons, with sub-millisecond time resolution. Among the three frequency ranges that we investigated, the lower two frequency ranges (gamma (30–80 Hz) and beta (12–30 Hz) range) showed similar network structure between cortex and hippocampus, but there were many significant differences between these structures in the high frequency range (100–1000 Hz). The high frequency networks in cortex showed short tailed degree-distributions, shorter decay length of connectivity density, smaller clustering coefficients, and positive assortativity. Our results suggest that our method can characterize frequency dependent differences of network architecture from different brain regions. Crucially, because these differences between brain regions require millisecond temporal scales to be observed and characterized, these results underscore the importance of high temporal resolution recordings for the understanding of functional networks in neuronal systems.

## Introduction

Understanding the detailed circuitry of neuronal networks is one of the major goals of neuroscience. Emergent properties at the systems level only come through the coordinated activity of large numbers of inter-connected neurons. Therefore, one must understand connectivity among neurons. However, the term ‘connectivity’ has several meanings. For example, there is a distinction between anatomical connectivity and functional connectivity. Anatomical connectivity describes whether or not neurons are physically (synaptically) connected; functional connectivity describes whether or not neurons have correlated activity. Even if neurons are anatomically connected with each other, if they don’t fire together, they will not have functional connectivity. Even if neurons do not share synapses, they could still be functionally connected if they receive common modulatory input. As a final distinction, the term ‘effective connectivity’ is also used to differentiate mere correlation from directed causal influence [1], [2], but we will not distinguish these two terms and will instead refer to them both as functional connectivity.

The analysis of network connectivity (network science) has been successfully applied to networks of macroscopic brain regions [3]–[5]. Studies of functional networks composed of individual neurons (referred to as “microscopic” networks) have been limited until recently by recording technology. Optical recording methods, such as calcium imaging [6], [7], and electrophysiological methods, such as large-scale multielectrode-array technology [8]–[11], have made it possible to simultaneously record the spiking activity from hundreds of neurons, a number sufficient for the application of graph-theoretic approaches.

There have been a few graph-theoretic studies of functional networks among hundreds of neurons using calcium imaging as reviewed in [2]. Based on these works, the network structure seems to be scale-free in the hippocampus [12], [13], or at least has small-world attributes [14]. However, these studies were conducted at relatively low temporal resolution (∼50 ms) and thus the fine temporal structure of the correlations (∼1 ms) has not yet been investigated. Importantly, there are many studies that suggest that brain networks may utilize rhythms at different frequencies, in addition to the millisecond scale for synaptic communication [15]. For example, the gamma rhythm seems to play an important role in perception and visual processing in cat cortex [16] and the beta rhythm appears to play a significant role in visuomotor integration [17]. Interestingly, the physiological mechanisms for generating gamma rhythms and beta rhythms exist independently in hippocampal CA1 circuitry [18]. Another study suggests that fast gamma (∼90 Hz) and slow gamma (∼40 Hz) rhythms in the hippocampal CA1 region segregate the input source by frequency [19]. These synchronies in multiple frequency bands were summarized in [20]. Neural recording with submillisecond temporal resolution could therefore provide a detailed comparison of functional network structure across different temporal scales or (equivalently) frequency ranges.

To investigate functional connectivity across a wide range of temporal scales, we used a 512-channel multielectrode array system developed at the University of California, Santa Cruz [8]. This system provides 60 µm spatial resolution (60 µm electrode spacing), and 50 µs temporal resolution (20 kHz sampling rate). This temporal resolution is three orders of magnitude finer than the ∼50 ms resolution typically achieved with calcium imaging [21]. In addition, the system records the spiking activity of hundreds of individual neurons simultaneously [22], [23].

Here we propose a novel method for analyzing functional connectivity between neurons at different frequency ranges. In this method, we analyzed cross-correlograms of spiking activity between neuron pairs using a wavelet transform. The wavelet transform revealed the different temporal structures in cross-correlations, which allowed us to directly compare the functional network structures of hundreds of neurons in organotypic cultures of cortex and hippocampus.

## Materials and Methods

### Ethics Statement

All neural tissue from animals was prepared according to guidelines from the National Institutes of Health and all animal procedures were approved by the Indiana University Animal Care and Use Committee (Protocol number: 12-015) as well as the Animal Care and Use Committee at the University of California, Santa Cruz (Protocol code: Litka 1105).

### Organotypic Culture Preparation

Organotypic cultures were prepared as previously described [22], [23]. Briefly, brains from postnatal day 6 (P6)-P7 Black 6 mouse pups of either sex were removed under a sterile hood and placed in Gey’s balanced salt solution for 60 minutes at ice cold temperature. After 30 minutes, half the solution was changed. Brains were next blocked into ∼5 mm^3 ^sections containing dorsal hippocampus and somatosensory cortex. Blocks were then sliced to a thickness of 400 µm using a vibrating blade microtome (Leica VT1000 S). The angle of the sections was closest to that of a coronal section, but the lateral side of the plane was advanced by 15 degrees in the anterior direction, so that both transverse sections of hippocampus and somatosensory cortex were included in the same tissue. These transverse sections are thought to preserve more of the hippocampal synaptic connectivity within the plane of the slice [24]. Each slice was put on a circular piece of filter paper (∼6 mm diameter), and grown in culture medium (50% minimum essential medium, 25% horse serum, 25% Hank’s balanced salt solution, 5 mg/ml D-glucose, 1 mM L-glutamine, 5 U/ml penicillin-streptomycin) in a heated (37°C), CO_2_ enriched (5%) incubator for 2–4 weeks.

### MEA Electrophysiology and spike-sorting

As mentioned above, all recordings were performed on a custom-made 512-electrode array system [8]. The flat electrodes were 5 µm in diameter and spaced 60 µm apart in a hexagonal lattice. The recording area was a 0.9 mm by 1.9 mm rectangle. Cultured brain tissues were gently placed on the electrode array using tweezers to hold the filter paper such that the tissue side was facing down and either the cortex or the hippocampus was centered on the array. Typically, the cortex was larger than the size of the array, and the short side (0.9 mm) of the array spanned across 70–80% of the thickness of the cortex. The hippocampus was smaller than the array, covering approximately ∼70% of the active area of the array. A small harp (∼1.3 g) with fine mesh (160 µm pore size) was placed on the filter paper on top of the tissue in order to ensure better contact between the tissue and the array. The tissue was perfused with oxygenated (95% O_2_/5% CO_2_) culture medium at a flow rate of 3 ml/min. After waiting for 30 minutes to allow the tissue to develop stable spiking activity, extracellular signals were recorded for 60 minutes on each of the 512 electrode channels at a sampling rate of 20 kHz. Raw waveforms were then spike-sorted with a well-established method developed by Litke et al. [8], with slight adjustment of the parameters for cortical brain slices. Briefly, signals that crossed a threshold of 8 SDs were marked, and the waveforms found at the marked electrode and its six adjacent neighbors were projected into five dimensional principal component space. A mixture of Gaussians model was fit to the distribution of features based on an expectation maximization algorithm. Duplicate neurons, neurons that had refractory period violations, and neurons with too few spikes (less than 100 spikes/hour) were excluded from further analysis.

### Immunohistochemistry and imaging

After electrophysiological recording, the cultures were fixed in 4% paraformaldehyde for 20–30 minutes, then stored in phosphate buffered saline with 0.05% sodium azide at 4°C. Cultures were washed three times with tris-buffered saline (TBS), then immunostained free-floating, ensuring that the tissue side faced upward (filter paper down). Cultures were blocked with 10% normal goat serum and permeabilized with 0.5% Triton-X for 2 hours, then washed with TBS and incubated with primary antibody (Neuronal nuclei (NeuN) – Chemicon Millipore (cat #MAB377) mouse monoclonal IgG1 anti-NeuN 1∶1000) overnight at 4°C on an orbital shaker. Cultures were then washed with TBS and incubated with secondary antibody (Molecular Probes Alexa Fluor 555 goat anti-mouse IgG 1∶1000) for 2 hours at room temperature on an orbital shaker. Next, cultures were washed with TBS, DAPI stained for 10 min (Invitrogen D21490/DAPI-Fluoro-Pure Grade, 300 nM working solution), and washed again with TBS. Tissues were mounted on superfrost/Plus slides, then coverslipped using Fluoromount-G (Southern Biotech) and allowed to dry overnight before imaging. Cultures were imaged on a Leica Spot Scanning SP5 confocal microscope using a 20x/0.5 objective lens; gain and offset were optimized to the z-plane of highest-intensity staining for each culture. Brightfield and fluorescent overlaid images were tiled in order to capture the entire hippocampus and filter paper marks, necessary to orient the placement of the culture on the 512-electrode array.

### Identification of hippocampal neurons

**Figure 1:**
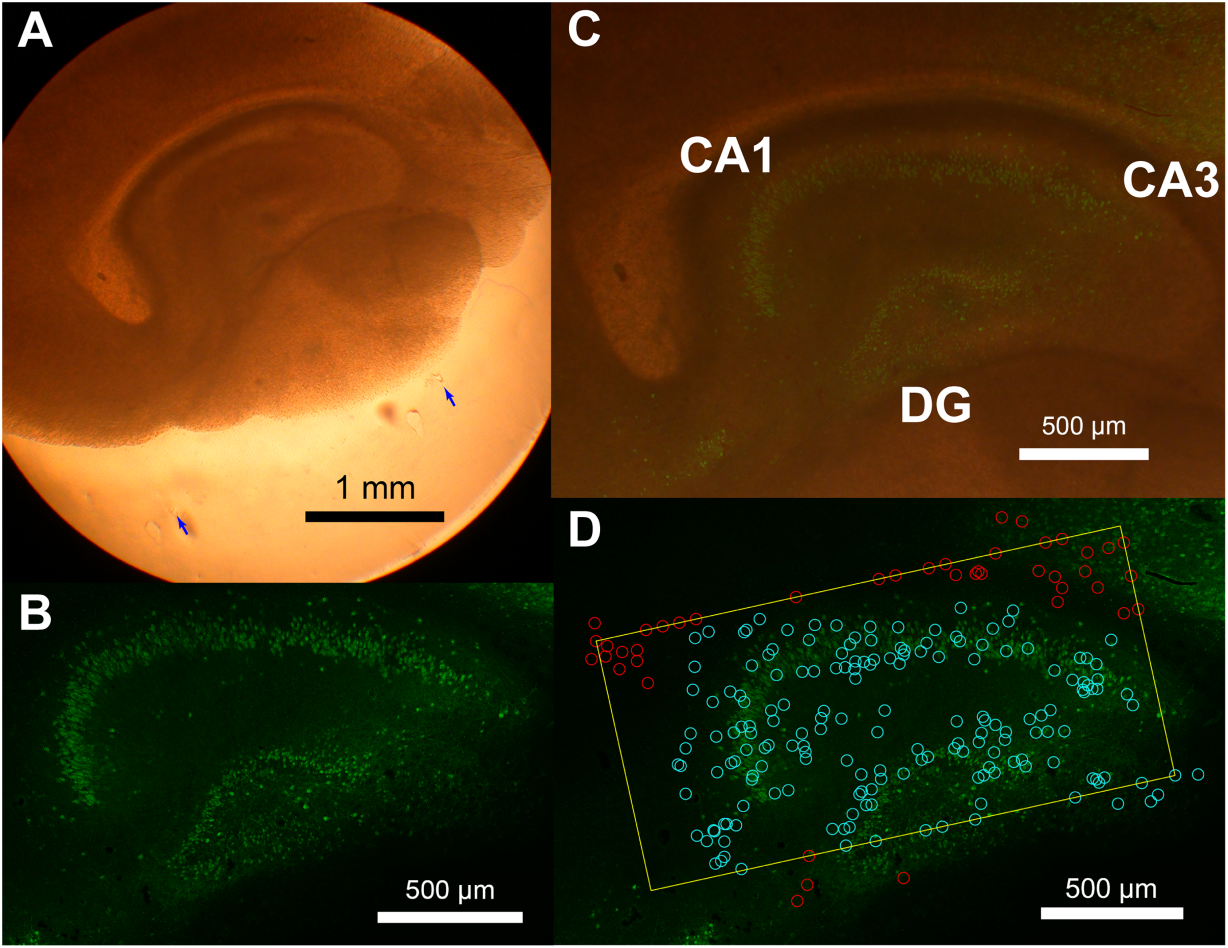
Organotypic culture preparation and photo overlay. Photographs of cortico-hippocampal organotypic cultures. Tissues are grown on a filter paper. A: A bright field image of an example organotypic culture at DIV1. The hippocampal structure is visible without staining. Blue arrows indicate the location of markers. B: NeuN staining of the culture after data taking and tissue fixation at DIV16. There are missing neurons in CA3 as consistent with a previous report (Zimmer and Gähwiler, 1984), but the overall layer structure is well conserved. C: Overlaid photograph of A and B. Relative position is adjusted by aligning the two markers on the permeable filter paper. Positions and dimensions of the hippocampal structures are well conserved during the incubation period. D: Overlaid photograph of B, the outline of the array (yellow rectangle), and the estimated locations of the recorded neurons. Light blue circles are manually identified hippocampal neurons (see ‘Identification of hippocampal neurons’ subsection), and red circles are neurons recorded outside the hippocampal structure. Locations of the recorded neurons match with the granule cell layer and the cell body layer.

Because the slice cultures contained portions of cortex and hippocampus, we sometimes recorded neurons from both brain regions. When slice cultures grow, the distinction between brain regions can often become blurred under bright field microscopy, but the gross structures of cortex and hippocampus are still preserved and can be observed by immunohistology (Figure 1B). In order to identify hippocampal neurons, we compared the photographs of the cultures before and after they were incubated. Photographs of the tissues were taken on the first day in vitro (DIV1) and then 2–4 weeks later (DIV14-28) after recording with the electrode array. The filter paper on which the tissue was placed was marked by two small cuts (Figure 1A, blue arrows) to allow the positions from the two photographs to be aligned. Aligned photographs suggest that the structures, locations and dimensions of hippocampal cell body layers (dentate gyrus (DG), CA3, and CA1 as labeled in Figure 1C) were well preserved after more than two weeks of the incubation period. The positions of the neurons were then estimated by fitting two-dimensional Gaussian distributions to the strength of the average signals on the electrodes (electrophysiological images [8]). Estimated neuron positions were then plotted on top of the DIV1 photographs (Figure 1A) to compare with the underlying anatomical structures. Only neurons that were within the hippocampal region on the photograph (Figure 1A) were retained for further analysis of the hippocampal network structure. Locations of the identified hippocampal neurons matched well with the cell body layer of the stained hippocampus (Figure 1D). For cortical recordings, we did not categorize neurons because the cortex was large enough to cover the entire array and there were no neurons from other structures present.

### Cross-correlation

Several different definitions of cross-correlation have been used in the literature. We chose to use the cross-correlation histogram (CCH), which is a histogram of time differences of spikes for a pair of neurons. The CCH between neuron *I* and neuron *J* is defined by the following equation:
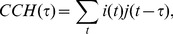
(1)where *i*(*t*), *j*(*t*) were the binary states of neurons *I* and *J* at time *t*. This binary state was defined to be 1 or 0, indicating a spike or no spike, respectively, in a time bin of width 50 or 500 µs centered at time *t* (see Table 1). Several methods for normalizing cross-correlations have been proposed in the literature [25]–[27]. However the final results are independent of the choice of normalization because we used a Monte-Carlo based method for our significance test. The details are described below in the ‘Network Analysis’ section.

**Table 1:** Parameters for wavelet transform.

	Timebin size	TransformedWindow(time bin centers)	Number oftime bins	Window forpeakidentification	CoveredFrequency	Number offrequency bins
Scale 1	50 µs	±70 ms	2801	±20 ms	20–1000 Hz	101
Scale 2	500 µs	±700 ms	2801	±200 ms	2–100 Hz	101

When two or more neurons are near the same electrode, their spike waveforms can interfere with each other, and the overlapped spikes are not sorted well. In such a situation, the cross-correlation can have an artifactual trough near the origin. Less frequently, when spikes from a neuron are recorded on multiple electrodes, a small fraction of these spikes can be misidentified as originating from another neuron. This creates a very sharp peak (∼50–100 µs width) in the cross-correlation near τ = 0. To prevent these artifacts from being identified as significant signals, we linearly interpolated a ±1 ms segment from the average cross-correlation from −1.5 to −1.0 ms and from 1.0 to 1.5 ms when the physical distance between neurons was closer than 180 µm (3 interelectrode distances).

### Wavelet Transform

**Figure 2:**
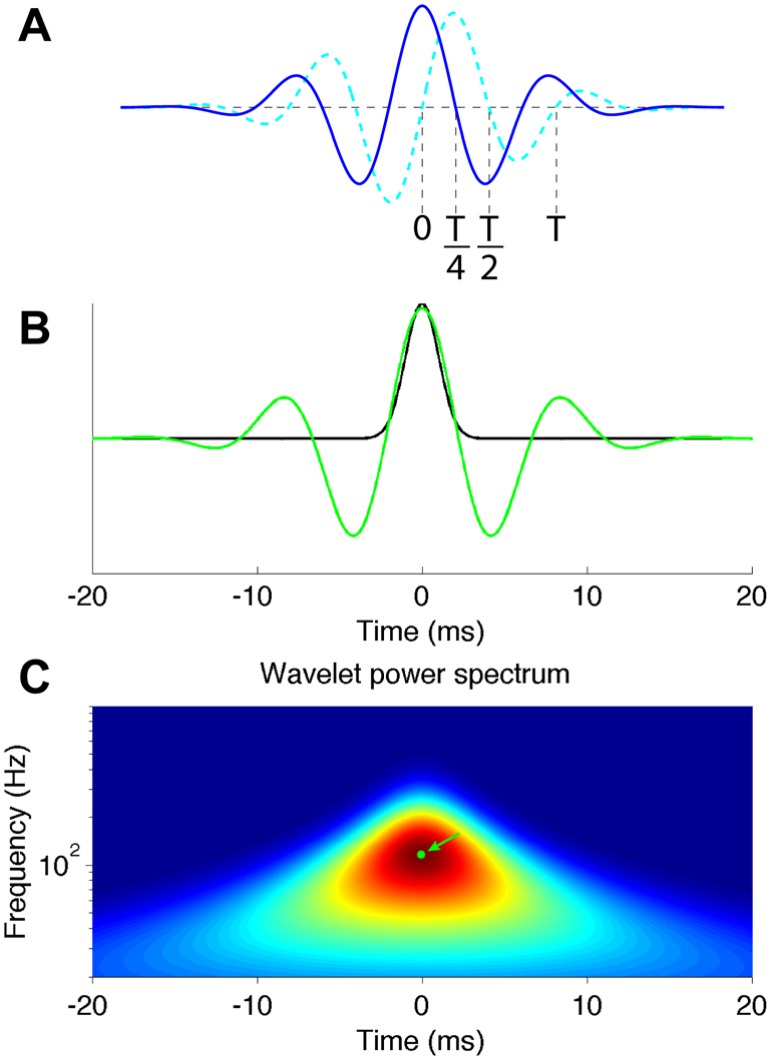
Example of a wavelet function and peak identification. A: An example of a complex Morlet function. The solid blue line is the real part; the dashed cyan line is the imaginary part. *T* is the Fourier period of the wavelet function. B: A zero-centered Gaussian peak with σ = 1 ms (black line) and the real part of the wavelet function that gives the maximum power (green line). The Fourier frequency and the delay time of the wavelet function were given by the peak in the wavelet power spectrum (C, arrowed green dot). C: Wavelet power spectrum of the Gaussian function defined in B. The wavelet power spectrum has a peak at time = 0 and frequency ∼116 Hz. This gives the approximate relation between the Fourier period and the Gaussian width: T ∼8.6σ.



**Figure 3:**
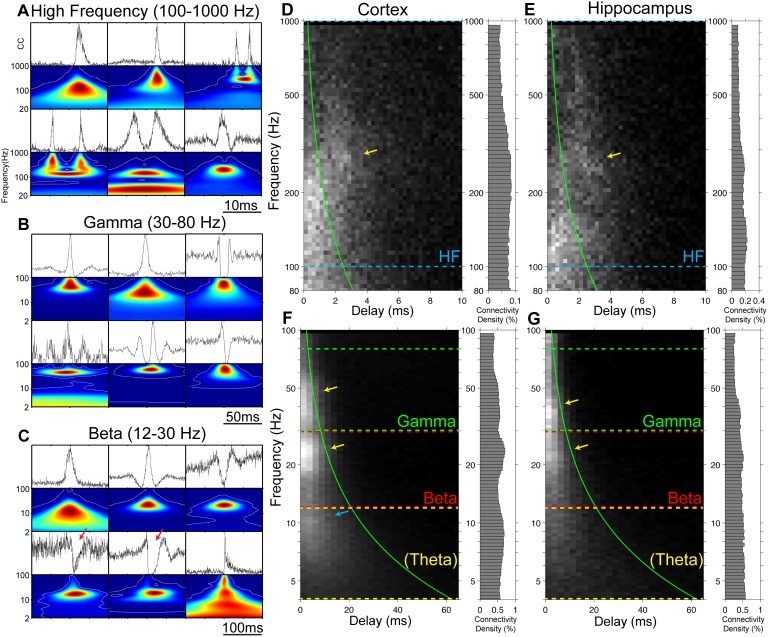
Examples of cross-correlations and their wavelet transforms. A–C: Six example cross-correlations and their wavelet transforms from each frequency range. Top of each panel is the cross-correlations of neurons. All these types of structures in the cross-correlation plots are observed both in cortex and hippocampus. The wavelet power spectrum of each pair is shown right below the cross-correlation. Hot colors indicate stronger power and cool colors indicate weaker power. White contours in the power spectrum indicate significance thresholds (See Materials and Methods). Note the wide variety of shapes that can be observed in each frequency range. A: Examples from high frequency connectivity. Typically, a single peak in cross-correlation was observed. Multiple peaks and oscillatory shapes were observed less frequently. Many connections showed a peak offset from zero (also see D, E). B: Examples from gamma frequency connections. Single peaks and troughs, and oscillatory shapes were the most common. C: Examples from beta frequency connections. Single peaks and troughs, and oscillatory shapes were found. Signs of inhibition on one of the sides also can be found (red arrows). D–G: The distribution of the delay and the frequency of the peaks of wavelet power spectra from cortex (D, F) and hippocampus (E, G) in scale 1 (D, E) and scale 2 (F, G). Examined frequency ranges were bounded by colored dashed lines. The right panel on each plot is the connectivity density at each frequency. Solid green curves indicate 1/4 of the Fourier period of the wavelet function. At this time delay, the primary peak of the wavelet function does not overlap with t = 0 (Figure 2A). If the delay is larger than this value, the connection is considered as ‘directed (delayed)’. Broadly tuned clusters of connections were observed in both scale 1 and scale 2 (yellow arrows), which motivated us to set three frequency ranges: high frequency (100–1000 Hz), gamma (30–80 Hz) and beta (12–30 Hz). A peak in the theta band (4–12 Hz) was observed in the cortex (F, blue arrow), but not in hippocampus (G) (see the ‘Cross-correlations and wavelet transforms’ subsection).

The wavelet transform is a widely accepted method for analyzing time series data and is especially useful when there are non-stationary oscillations [28]. In neuroscience, wavelets have been used to analyze continuous signals such as local field potentials (LFPs) and electro-encephalogram (EEG) recordings [29]–[31]. The wavelet transform has not been widely used as a method to analyze single unit spike trains (but see [32], [33]). As far as we know, no one has applied wavelet analysis to cross-correlation functions to assess functional connections, making ours a novel approach. We adopt the mathematical framework and notation used by Torrence and Compo [28]. The continuous wavelet transform is calculated by convolving a mother wavelet function with the signal (here, the cross-correlogram). We have chosen a complex Morlet function as the mother wavelet function:
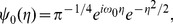
(2)where *ω_0_* is the non-dimensional frequency of the wavelet function, here taken to be 4. An example of the wavelet function is shown in Figure 2A. The wavelet transform of the cross-correlogram is given by convolving a scaled, translated and normalized version of this mother wavelet function with the cross-correlogram:

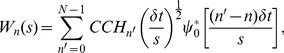
(3)where the (*) indicates the complex conjugate, *δt* is the time step of the CCH (we have summarized the parameters for the wavelet transform in Table 1), and *N* is the number of time steps in the cross-correlogram. As seen in equation (3), the wavelet transform is given as a function of both scale (*s*; width of the wavelet function, which corresponds to the Fourier period) and time (*n*). In order to minimize the influence from the edge of the data, we padded both ends of the data with the average of 100 bins at the edge of the CCH such that the total number of bins is a power of 2. Only limited segments of the wavelet transform are used in the following analysis (Table 1), so these edges did not affect our results. The wavelet power spectrum is calculated by taking the absolute square of each point of the wavelet transform. The wavelet power spectrum can be used for identification of both simple peaks and oscillations. An illustrative example of peak identification is shown in Figure 2B, C. Example cross-correlograms and their wavelet power spectra from data are shown in Figure 3A–C. The same framework can be used in different frequency ranges by merely changing the binning size. Here we have used only 2 different scales for our network analysis. The frequency bins were set to equal size in log space.

### Connectivity analysis of the wavelet power spectrum

Once the wavelet power spectrum was calculated, it was subjected to a significance test. The significance test was done by comparing the value of the wavelet power of the data with the value of the wavelet power from a noise distribution. To avoid frequency dependent biases, we chose white noise as our noise distribution, which has a flat power spectrum across all frequencies. Although the analytical form for the wavelet power spectrum is known for a Gaussian white noise time series [28], the equivalent distribution for Poissonian white noise – the type of noise of our cross-correlations – is not known. Therefore, for this study, the significance threshold was calculated with a Monte-Carlo method by generating white noise cross-correlograms many times. The threshold values were determined as a function of frequency and the number of spikes in the cross-correlogram. Because it is computationally infeasible to generate a threshold for every possible number of spikes, we generated a look up table of threshold values for different numbers of spikes at 10*^k^*, where *k* = 0.0, 0.1, 0.2, … 6.0. We linearly interpolated the results between these numbers. For each unique number of spikes, random white noise cross-correlograms were generated by assigning random timing in the transformed window to each spike, creating Poisson statistics at each bin. These cross-correlograms were generated 10^5^ times, and the wavelet power spectrum for each of the correlograms was calculated. Then, the maximum values of the wavelet power spectrum over time were evaluated at each frequency. This produced a distribution of 10^5^ maximum values at each frequency. Finally, a *p* = 0.001 threshold was enforced using the 100th highest maximum value at each frequency.

When analyzing the wavelet transforms of the experimental data, peaks (Figure 2C, green dot, arrowed) were defined as any point that had a value greater than the 8 surrounding points (all the pixels that surround one pixel in discrete 2-D space) and were detected from the regions of the power spectrum where their value exceeded the threshold. Thus each cross-correlogram could have multiple peaks at different frequencies and times. If the peaks occurred in different frequency ranges, they were treated as different connections. In this manner, functional connections between neurons could be evaluated at any desired frequency band. The directionality of the connection depended on the time offset of the correlogram peaks. If the peak position was more than 1/4 of the full Fourier period away from the center of the cross-correlogram, the connectivity was considered as delayed; otherwise, we considered it non-delayed. The shift of 1/4 of the full Fourier period is where the primary peak of the real part of the wavelet function does not overlap with t = 0 (Figure 2A). For example, a simple Gaussian peak is considered as a delayed connection when the time offset of the Gaussian is more than ∼2.2σ, because of the relationship between the Fourier period and the Gaussian width (Figure 2C, caption). Delayed correlograms were regarded as cases of unidirectional functional connectivity; non-delayed correlograms were considered to be cases of bidirectional connectivity.

### Network analysis

The strength of wavelet analysis lies in its ability to categorize neuronal correlation across different frequency ranges. Based on the distribution of the observed wavelet power peaks (see Figure 3 in the Results section), we chose 4 different frequency ranges in our work (Table 2). The first category was high-frequency connectivity (HFC), which we considered to be from 100 Hz to 1000 Hz. In most cases, peaks in correlograms with less than 5 ms width fell into this category. There were a number of neuron pairs that showed cross-correlations that produced HFC (See Results). The second category was gamma-frequency connectivity (GFC), which we considered to be from 30 Hz to 80 Hz. Both hippocampus and cortex are known to produce gamma rhythms in LFPs [34]–[39]. Even though we do not record LFPs, spikes of the neurons can synchronize through them. The third category was beta-frequency connectivity (BFC), which we considered to be from 12 Hz to 30 Hz. This rhythm has also been widely reported in the literature [39]–[41], although the beta oscillations in mouse hippocampus might be limited to in-vitro preparations [42]. The fourth category was the theta-frequency connectivity (TFC) (4–12 Hz) – another well-known rhythm in both cortex and hippocampus in the literature [15], [43], [44]. The results from the TFC largely overlapped with those from BFC, except for the decay length of the connectivity density. We report the results from the theta frequency range in the subsections where we observed differences from BFC.

**Table 2:** Parameters for the connectivity analysis.

Type of connectivity	Taken from	Frequency range
High frequency (HFC)	Scale 1	100–1000 Hz
Gamma (GFC)	Scale 2	30–80 Hz
Beta (BFC)	Scale 2	12–30 Hz
Theta (TFC)	Scale 2	4–12 Hz

We assigned the names of these frequency ranges based on common oscillations observed in continuous waveform data such as EEG or LFP recordings. The oscillatory shapes that were observed in Figure 3B, C could be correlated with such oscillatory electric fields. Examples of phase-locking between spikes and fields have been observed in many regions of the brain including cat visual cortex [38] and hippocampus [45]. However, this does not mean our wavelet peaks in these frequency ranges are always associated with field oscillations. Our analysis found simple peaks and troughs, as well as other non-oscillatory shapes, in both gamma and beta ranges (Figure 3), which may not be related to gamma and beta rhythms. If we also recorded LFPs, we could potentially study the relationship between the LFPs and the spikes. However, the relationship between the extracellular LFPs and spiking activity is still an active area of study [46], [47]. Our primary focus in the present study is to construct and evaluate networks of individual neurons. Thus we did not try to assess the relationship between the constructed networks and the extracellular LFPs.

Previous work has shown that there is a hidden bias that produces potentially misleading results in network measures when the networks have different numbers of nodes (neurons) and edges (connections), as described in [48]. In an attempt to mitigate this problem, we randomly subsampled 100 neurons from each tissue, and evaluated each network measure as a function of connectivity density. (Connectivity density is the fraction of connected pairs of neurons among all possible pairs of detected neurons.) In this way, we can compare networks with similar numbers of nodes and edges. We repeated subsampling with 100 different sets of randomly-chosen neurons, and calculated the mean and root mean square of each network measure as a representative value, and its associated error, for that one tissue. The tissues that had less than 100 neurons were excluded from the analysis (18/34 in hippocampus and 2/24 in cortex). Connections were ordered by significance, which was the ratio of the actual power of the peak to the threshold power. Then we evaluated the network properties as a function of the connectivity density from 0.5 to 2.0%.

Once the connections were selected, they were treated as binary connections (connected or not connected). We used connectivity densities up to 2%, although there is previous work that suggests a higher connectivity density (28.8%) is present for adjacent neurons in hippocampal culture [14]. There are several reasons why we expect significantly lower connectivity density in our preparations. First, we collected neurons from a large area (0.9 mm×1.9 mm). Therefore, many of the neurons were physically separated by large distances. Indeed, we observed higher connectivity density when the inter-neuron distance was short (see Results). Second, we measured functional connectivity from the cross-correlation of the spikes. Due to reduced statistics, it is possible that we were not able to detect as statistically significant the connections with limited strength. Indeed, sometimes we had tissues that had low connectivity; 13% of the subsampled networks had less than 1% connectivity density; 30% had less than 2%. Even if the subsampled networks had smaller connectivity density than required, we included those networks in the analysis (i.e. the data point for 2% connectivity density includes 30% of networks with connectivity density smaller than 2%. The connectivity density value specified in the figure should be understood as an upper limit of the connectivity density.). When the connectivity density for a given frequency range and data set was less than the specified value, the connectivity densities of the other frequency ranges for the same data set were adjusted to the connectivity density of the frequency range with the lowest connectivity density value in order to avoid a bias due to different connectivity densities in different frequency ranges. We did not simply reject subsampled networks with smaller connectivity density because doing so would cause a bias towards the inclusion of high-degree nodes.

We also measured the connectivity density as a function of distance between the neurons. We measured the distance between a pair of neurons from the estimated locations of the cell bodies (see ‘Identification of Hippocampal Neurons’). We counted the number of pairs as a function of inter-neuron distance in 50 µm bins, and then calculated the probability to observe connected pairs in each bin. Results were averaged over all the cultures, and the mean and the SEM were calculated. We fit the following exponential function with a constant baseline to the resulting probability function:
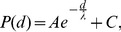
(4)where *A*, *λ*, and *C* were fit parameters, and *d* is the physical distance between the neuron pair. Fitting and evaluation of the confidence interval of the fit parameters were performed using the method of least squares in the Matlab Statistics Toolbox.

Once the connections are defined, we measured the network topology of our recorded neurons. “Network topology” refers to the arrangement of the elements – nodes and edges – of a given network. Various network measures were proposed to evaluate network properties as summarized in Box 2 of [49]. Among these measures, we chose relatively simple measures that could be easily compared across different frequency ranges and brain regions: the degree distribution, clustering coefficients, efficiency, and assortativity. These measures were evaluated as a function of connectivity density using the Brain Connectivity Toolbox [50]. Because of the complexity of the comparison across different frequency ranges and brain regions, a thorough analysis of motif structures, module structures, and identification of hubs was beyond the scope of this paper. Here are descriptions of the network measures we used:

#### Degree distribution

The degree distribution of the nodes – the probability distribution of the number of connections per neuron – was calculated from subsampled networks of 100 neurons with a 1% connectivity density, which was in the middle of the range of the connectivity densities we investigated. We will only report our results for output degree, as we achieved the same basic results for the input degree (data not shown). With regard to subsampling, it should be mentioned that scale-free networks are known to not produce a scale-free degree distribution when subsampled [51]. In order to simulate this effect, a subsampled scale-free degree distribution was calculated by subsampling 100 neurons from a 1000 neuron Barabasi-Albert scale-free network produced by preferential attachment at the same 1% connectivity density. [52]. The Barabasi-Albert networks were generated 1000 times and, for each network, the subsampling process was repeated 100 times. We also tried subsampling from 500 neurons and 10000 neurons instead of 1000 neurons; the resulting distributions were the same (data not shown). The degree distribution for the random network was given by the binomial distribution [53], and an exponential probability density distribution was used to create the degree distribution for the exponential network. The parameters for these three networks can be uniquely determined if the number of neurons and the connectivity density are known. The equations and parameters used to generate model degree distributions are summarized in Table 3. Also, we measured the number of disconnected nodes (nodes with zero input and output degrees).

**Table 3:** Probability density functions for the model degree distributions.

Type of model network	Probability density function
Random (binomial)	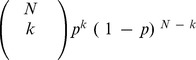
Exponential	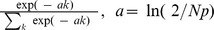
Scale-free (Barabasi-Albert)	N/A (see Materials and Methods)

*k* is the degree, *N* is the size of the network (the number of neurons; *N* = 100, in our networks). *p* is the connectivity density (*p* = 0.01 for Figure 7).

#### Clustering coefficient

The clustering coefficient measures the tendency of connections among network nodes to cluster together locally. If the neighbors of a node (those nodes connected to a given node) are themselves connected together, the clustering coefficient is high. The mathematical definition of the local clustering coefficient is as follows.
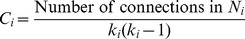
(5)where *N_i_* is a subnetwork consisting of neighbors connected to neuron *i* (but does not include neuron *i*) and *k_i_* is the number of neurons in subnetwork *N_i_*. The local clustering coefficient is calculated for the neurons that have *k_i_*≥2. A global clustering coefficient is calculated by averaging the local clustering coefficients of the neurons that have *k_i_*≥2. We used only the global clustering coefficient in our analysis.

#### Efficiency

The efficiency of the network is defined as an average of the inverse of the shortest path length. Here, the path length is defined as the number of edges that must be traversed to go from one neuron to another, not the physical distance between neurons. The average shortest path length is also a widely used measure, but the efficiency, the mean of the reciprocal of the path length, is not divergent in the case of disconnected nodes. (The path length of a disconnected pair is defined as infinity.) While it is true that this problem can be avoided by considering only ‘connected’ parts of the network, this approach would throw away a large number of nodes when the connectivity density is low. This measure is strongly dependent on the connectivity density, and thus it is quite important to compare at the same connectivity density, as we have attempted to do.

#### Assortativity

Assortativity measures the tendency of nodes to connect to other nodes that have a similar degree. (The degree of a node is its number of connections.) If high degree nodes preferentially connected to other high degree nodes, the assortativity is positive. Many social networks have positive assortativity, and many technological and biological networks have negative assortativity [54]. Assortativity is defined as the Pearson correlation coefficient of degree for connected nodes. The most convenient form of the assortativity is:
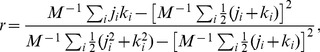
(6)where *j_i_*, *k_i_* are the degrees of the vertices at the end of the *i*th edge, *M* is the number of edges, and *i* = 1, …, *M* [54].

The significance tests for the comparisons of these network measures were done by two-tailed Student’s t-test. The network measures were checked prior to the application of the t-test to ensure they were roughly Gaussian (data not shown).

### Data sharing

We welcome enquiries concerning data sharing of our neural spiking activity data.

## Results

### Electrophysiological properties of the cultures

**Figure 4:**
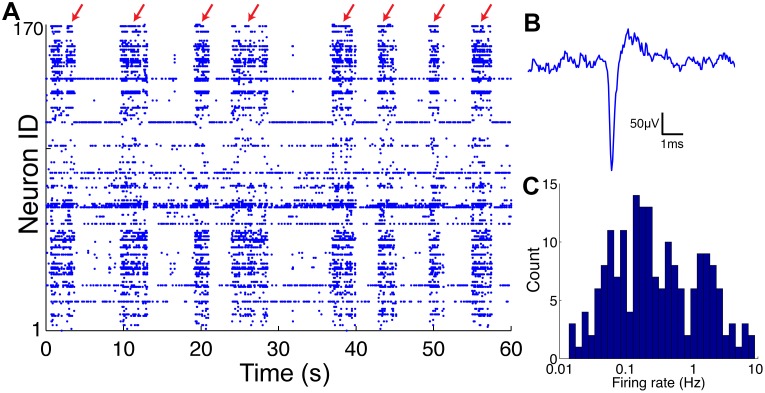
Raster plot, spike waveform, and firing rate distribution of a representative hippocampal recording. A: An example raster plot from one of the hippocampal tissues. There were events during which many of the neurons synchronously raise their firing rates (Red arrows, so called ‘network bursts’. See ‘Electrophysiological properties of the cultures’). These events typically lasted for seconds, and were observed in all of the hippocampal and cortical recordings. B: A representative extracellular spike waveform recorded with our system. The full width at half max was ∼0.3 ms. C: The firing rate distribution of the tissue in A.

In total, 59 cortico-hippocampal tissues were used for recordings. 25 tissues were used for cortical recordings; 34 tissues were used for hippocampal recordings. The average number of neurons found in each tissue of cortex and hippocampus was 315±127 and 118±51, respectively. The firing rate distribution is shown in Figure 4C. The firing rate for all the neurons in cortex and hippocampus were 10^−0.16±0.64 ^Hz and 10^−0.44±0.68 ^Hz, respectively. Cortical neurons had a higher average firing rate, and also a larger number of neurons were found in cortical recordings than hippocampal recordings. This smaller number of neurons in hippocampal recordings could be explained by the fact that the hippocampi were not large enough to cover the entire recording array (0.9 mm×1.9 mm); on average the hippocampus covered ∼70% of the array. The tissues that had less than 100 neurons (2 in cortex, 18 in hippocampus) were excluded from further analysis because they had too few neurons for meaningful network analysis. Non-stationary synchronous activity (so-called ‘network bursts’ [55], [56]) were seen in all the tissues in our recordings (Figure 4A). One cortical tissue that showed unusual network bursts that lasted ∼100 seconds was excluded from further analysis. Various types of bursts are summarized in [57]. In summary, 22 cortical recordings and 16 hippocampal recordings were used for further analysis.

### Cross-correlations and wavelet transforms

Representative cross-correlations and their wavelet-transforms are shown in Figure 3A–C. The example pairs were selected from the significant cross-correlations of both hippocampus and cortex. We measured the delay and the frequency of the peaks of the wavelet power spectra, and determined whether the correlations occur at specific delay or frequency ranges (Figure 3D–G). Large numbers of directed (delayed) connections were observed in the frequency range above 100 Hz in both cortex and hippocampus (Figure 3D, E; connections to the right of the green lines). In the scale with lower frequencies, we could observe peaks in the non-directed (non-delayed) connections at ∼40 Hz and ∼20 Hz both in cortex and hippocampus (Figure 3F, G; connections to the left of the green lines; peaks indicated by the yellow arrows). Another peak at ∼9 Hz, which falls in the theta frequency range (8–12 Hz), was observed in cortex (Figure 3F; blue arrow), but not hippocampus. It is known that hippocampal theta rhythm is dependent on afferent input [43], [58]. Presumably, the reason why we do not see the prominent theta rhythm of the hippocampus is the lack of afferent input in our organotypic cultures. The observed peak structures in Figure 3D–G motivated us to separate the frequencies into the 4 ranges (High Frequency, Gamma, Beta and Theta) described in ‘Network analysis’ in the ‘Materials and Methods’ section. We next describe the characteristics of the identified connectivity in each frequency range (except for TFC, which was similar to BFC).

Connections in the HFC (100–1000 Hz) range had very temporally precise cross-correlation. Peaks that had a time offset from zero were the most common in this frequency range (62% directed and 38% non-directed at 1% connectivity density). Troughs and oscillatory shapes were rarely observed in this frequency range.

Connections in the GFC (30–80 Hz) range had relatively more pairs that had zero-centered cross-correlation. Single peaks or single peaks with a little trough on the side were the most common cross-correlations. However, compared to HFC, more troughs and oscillatory shapes were observed. At this frequency range non-directed connections were the most common (24% directed and 76% non-directed at 1% connectivity density).

Connections in the BFC (12–30 Hz) range had even larger temporal structure. The properties of connectivity here were similar to GFC; peaks, troughs, and oscillatory shapes were all observed in this frequency range as well. One notable difference was that sometimes inhibition with relatively long duration from one neuron to another was observed in this frequency range. (Figure 3C, red arrows) Directed connections were found even less often in this frequency range. (17% directed and 83% non-directed at 1% connectivity density).

As mentioned in the ‘Network analysis’ subsection in Materials and Methods, we observe simple peaks and troughs in both the gamma and beta frequency ranges, which may not be related to field oscillations.

**Figure 5:**
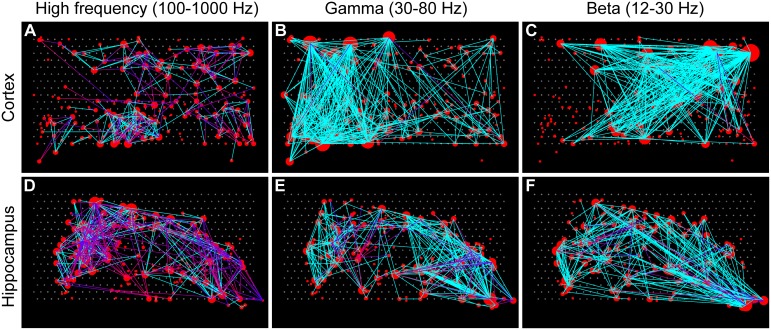
Example connectivity maps. Connectivity maps of one of the tissues from cortex (A–C) and hippocampus (D–F) at three different frequency ranges: high frequency (A, D), gamma (B, E), and beta (C, F). The locations of the red circles indicate the estimated locations of the neurons. The size of a circle indicates the number of functional connections (degree) of the associated neuron with other neurons. Lines with a color gradient from blue to red indicate directed (delayed) connectivity that goes from the blue end to the red end; solid cyan lines indicate non-directed (non-delayed) connectivity. The threshold value for delayed connections was set to 1/4 of the Fourier period of the wavelet function (see Materials and Method). Larger numbers of directed connections were observed in high frequency networks (A, D). One can see features such as the absence of high degree nodes and a shorter connectivity range in the high frequency cortex network (A), which are better quantified later.

Given these three frequency ranges, we can construct three different neural connectivity networks per recording. Example connectivity maps from both cortex and hippocampus are presented in Figure 5. This figure illustrates features of different structures in different frequency ranges and in different brain regions. For example, the differences of the degree distribution and shorter connectivity range in high frequency cortex compared to high frequency hippocampus are visible. These observed features are better quantified in the following section.

### Network structure differences across frequency ranges

**Figure 6:**
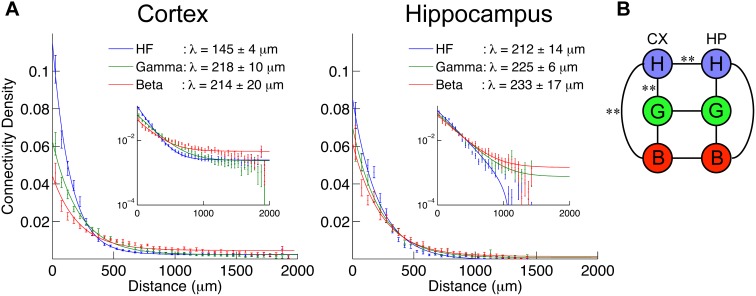
Decay of connectivity density over distance. A: Connectivity density as a function of distance. The distance was binned in 50 µm bins, and the connectivity density was evaluated at each distance. Error bars are SEM of all the cultures. Different colors represented different frequency ranges. Solid lines are exponential function fits to the data (see Materials and Methods). In the legend, the decay lengths of the function fits are displayed. Inset is the same figure plotted in semi-log space. Exponential functions fit the data nicely, and give us the decay length λ. Consistent with other network properties, we observed differences in different frequency ranges in cortex, but not in hippocampus. B: Results of significance tests for the decay length λ. The tests were done among different scales in the same structure, and different structures at the same scales (black lines). The significance was evaluated from the standard deviations of λ, assuming a normal distribution. One star signifies p<0.05 significance; two stars are p<0.01 significance. While we saw no significant differences in hippocampus, the high frequency range in cortex was significantly different from the other two frequencies.

The decay of the connectivity density over distance is presented in Figure 6. Exponential decay with a constant baseline fit well the experimental measurements, and the decay constant was estimated for each frequency range. Although the data are not presented in the figure, the decay lengths of the theta frequency range (4–12 Hz) networks were 301±43 µm and 237±30 µm in cortex and hippocampus, respectively. In cortex, we observed faster decay in the high frequency network and slower decay in the theta frequency network, but such differences were not observed in hippocampus. This measure in cortex was the only measure that showed differences between the beta networks and the theta networks.

**Figure 7:**
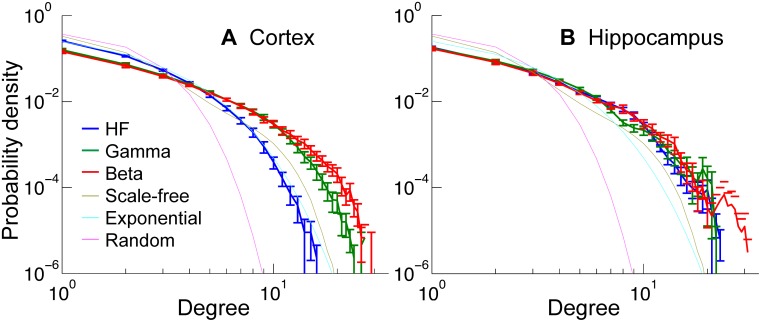
Degree distribution. Average degree distribution of all frequency ranges for networks set at 1% connectivity density. The thin lines are the degree distributions of simulated model networks (scale-free, exponential, and random networks). The degree distribution of the scale-free network was achieved by randomly subsampling 100 nodes from a 1000 neuron scale-free network. Because of the subsampling effect, the result from the scale-free network does not appear as a straight line in this log-log plot (see Materials and Methods). Error bars indicate the SEM of 25 and 22 data sets in hippocampus and cortex, respectively. A: Degree distributions of cortical networks. Gamma and beta networks had longer tails than a scale-free network, but the high-frequency network had a shorter tail than the other two. B: Degree distribution of hippocampal networks. All the frequency ranges had similar degree distributions, which had longer tails than a scale-free network.

Degree distributions of 100 subsampled networks are shown in Figure 7. The degree distributions of the model networks (random, exponential, scale-free) were also shown. None of the distributions from the data showed an acceptable match (>5% confidence level) to the model distributions. The closest match was the cortical HFC network to the exponential distribution (χ^2^_red_ = 2.0, ∼1% confidence level), but the others did not match at all (χ^2^_red_>10, <10^−15^% confidence level). The cortical HFC network showed a different degree distribution from that produced by GFC and BFC. It was the only network that had a shorter tail of the degree distribution than that produced by the simulated scale-free network. (Recall that this scale-free network degree distribution does not appear as a straight line because it contains 100 nodes that are subsampled from a 1000 node scale-free network [51], as described in Materials and Methods.) On the other hand, in hippocampus, all the frequency ranges showed similar degree distributions. The networks produced by all the frequency ranges examined had longer tails than those produced by the scale-free network.

**Figure 8:**
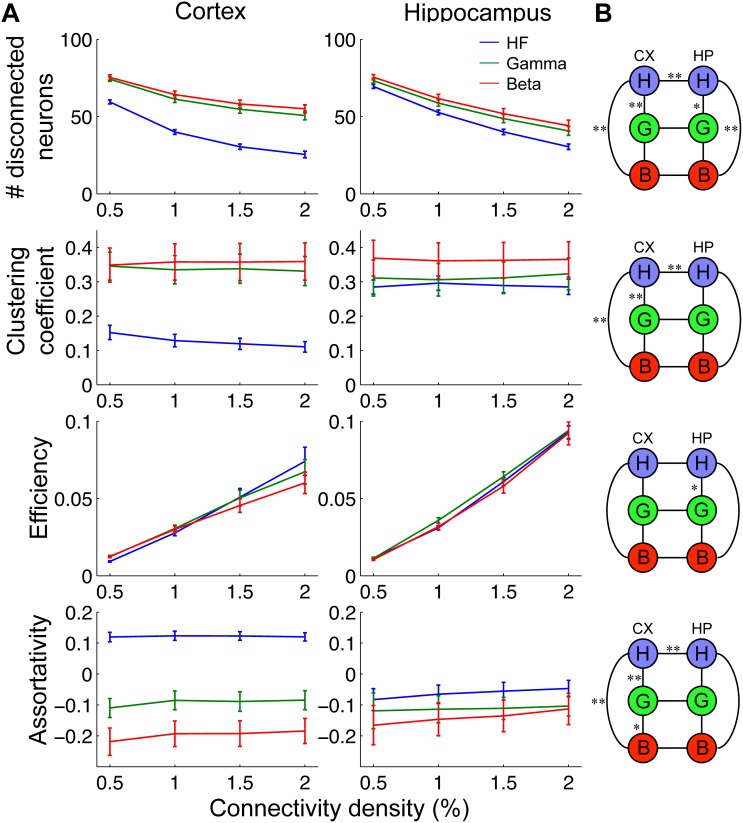
Graph theoretic measures in 3 different frequency ranges. A: Various network measures were determined for networks in different frequency ranges as a function of connectivity density. Error bars indicate SEM of 22 and 16 data sets in cortex and hippocampus, respectively. *Number of disconnected neurons:* These are the number of neurons with zero input and output degrees, in subsampled networks of 100 neurons. Both in cortex and hippocampus, high-frequency networks had a smaller number of disconnected neurons. The high-frequency cortical networks had a significantly smaller number than the hippocampal networks. The number of disconnected nodes may affect other network measures through changing the effective size of the networks (see Results). *Global clustering coefficient:* All the frequency ranges in hippocampus showed similar values. Note that the high-frequency cortical networks had lower values than all the other networks. *Network efficiency:* There were no differences with p<0.01 significance in this measure (see B). The values grew monotonically with connectivity density. *Assortativity:* Hippocampal networks showed slightly negative values in all the frequency ranges. The low frequency networks showed lower assortativity. In cortex, the values were significantly different in each frequency range. The high-frequency networks showed positive assortativity unlike all the other networks. B: Results of significance tests (two tailed Student’s t-test) of network measures at 1% connectivity density. The tests were done among different scales in the same structure, and different structures at the same scales (black lines). One star represents p<0.05 significance, and two stars represent p<0.01 significance. Note that significant differences were observed in different scales in cortex, and between cortex and hippocampus especially at the high frequency range, but fewer significant differences were observed between the different scales in hippocampus.

Multiple network topology measures showed differences in different frequency ranges and brain regions (Figure 8). The number of disconnected neurons (out of the subsampled networks of 100 neurons) was smaller for HFC both in cortex and hippocampus, but the number in cortex HFC was even smaller than in hippocampal HFC. Having a large number of disconnected nodes increases the effective connectivity density of the ‘connected’ part (neurons with any connections) of the network. The clustering coefficient was low for HFC compared to GFC and BFC for cortex, but there were no differences for hippocampus. There was not much difference observed in the efficiency across frequencies both in cortex and hippocampus. Assortativity was significantly different across frequency scales in cortex. For cortex HFC, assortativity had a consistently positive value, and in the other two scales it had negative values. Again, there were no differences across frequency ranges in hippocampus.

### Simulation of low resolution recordings

**Figure 9:**
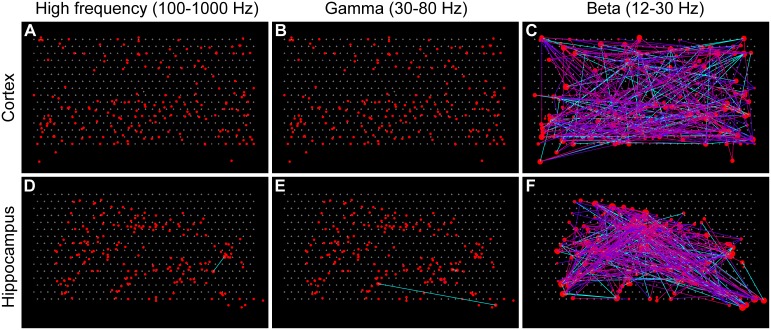
Connectivity maps with 50 ms jittering. The same threshold values as Figure 5 were applied to each connectivity map of the same data sets. Most of the connections in HFC and GFC were lost (A, B, D, E). Connections in the Beta frequency range (C, F) were different from the original connectivity maps (Figure 5C, F).

In order to simulate low time resolution recordings, we jittered the spike time data by a Gaussian distribution with a sigma of 50 ms. We used the same data sets as in Figure 5 as an example. The connectivity maps of the jittered data are shown in Figure 9. The significant threshold was the same as that of Figure 5. Most of the connections in HFC and GFC were lost by the jittering. Connections in BFC were largely different from the original connections shown in Figure 5. These results underscore the importance of high temporal resolution recordings.

## Discussion

### Summary of findings

We have shown that the wavelet transform can categorize pairwise neuronal cross-correlations into different frequency ranges. Some cross-correlation patterns suggested synaptic communication and some implied correlation due to other mechanisms, such as common drives, gamma/beta/theta rhythms, and long-term inhibitions. We have also shown that correlations were observed in 4 frequency ranges (HFC, GFC, BFC, TFC), and they can show different network structures. Among all the networks, the cortex HFC networks were the most different. They had positive assortativity, a smaller decay length, and a shorter tail of the degree distribution. The hippocampal networks at different frequency ranges showed less diversity. These results suggest that measurements of functional connectivity at multiple frequency ranges, especially with high temporal resolution recordings, are important to illustrate the differences of functional networks.

### Functional connectivity and anatomical connectivity

We have introduced a new method to obtain functional connectivity in different frequency ranges. What do these different frequency ranges say about the underlying synaptic connectivity in these tissues? It is difficult to determine from cross-correlation alone whether a pair of neurons is synaptically connected. However, because synaptic connectivity is known to have temporal scales of a few milliseconds, some researchers have argued that cross-correlograms with sharp peaks at short delays may indicate direct synaptic connections [59], [60]. In the present work, the majority of connections in this class would fall into the HFC category because of its temporal sharpness. Indeed such examples can be observed in HFC in Figure 3A. On the other hand, it is less likely that we can associate lower frequency range connectivity with direct synaptic connections because most of them were non-delayed. Again, we did not try to assess whether or not HFC was mostly due to monosynaptic connectivity, but it is reasonable to think that the functional network structure presented by HFC was closer to the structural synaptic connectivity than were the functional networks presented at the lower frequency ranges.

### Degree distribution in cortex and hippocampus

There are a few reports about the degree distribution of neuronal networks. In rat and mouse hippocampus, the degree distribution of the functional connections is scale-free [12] (calcium imaging; acute slices; cross-correlation connectivity; thousands of neurons; 50–150 ms time bins). In rat cortex, the degree distribution falls as fast as random networks [61] (simultaneous patch clamp; acute slices; monosynaptic connectivity; 6 pyramidal neurons). In cat visual cortex, the degree distribution also falls as fast as a random network [62] (extracellular electrophysiology; in-vivo under visual stimulation; maximum entropy connectivity; 10 neurons; 2 ms time bins).

In our preparations we measured the degree-distribution in 100-neuron networks. The degree-distributions of all the networks had longer tails than that of random networks (Figure 7). Cortex HFC had a relatively shorter tail compared to other networks, and was closest to the degree distribution of an exponential network. All the other networks had longer tails than all the model networks including a subsampled scale-free network. Differences between cortex and hippocampus were observed clearly in HFC. The degree distribution in cortex HFC (exponential) does not match with the previous reports (random). However, it is difficult to directly compare our results with these previous results due to differences of animal species and preparations, limitations of the number of neurons or temporal resolution, and definitions of connectivity. We are also uncertain whether we are able to expect that a simple model distribution matches our data without distinguishing different cell types, as different cell types may have different connectivity patterns.

We note that because the possible number of connections between neuron pairs is proportional to the number of neurons squared, our MEA, which has 512 electrodes, was able to collect a great amount of cross-correlation data. Thus, this detailed evaluation of the degree distribution is a new result that utilized the advantage of the large-scale multielectrode-array. We also note that MEAs with an even greater number of electrodes have recently become available [9], [10]. Brain slice experiments performed with these very-large-scale MEAs may potentially benefit from the computational methods described herein.

### Positive assortativity in cortex HFC

When Newman proposed ‘assortativity’ for the first time, he found that technological and biological networks typically show slightly negative assortativity while social networks show positive assortativity [54], [63]. Later on, examples of positive biological networks were found in macroscopic human brain studies [64], [65]. One study suggested that networks with negative assortativity enhance stability of synchronization [66]; other studies suggested positive assortativity enhances resilience to targeted attacks [54], and robustness to noise [67]. Another study suggests that the network will naturally evolve to have slightly negative assortativity unless there is a specific mechanism for making positive assortativity [68].

In our results, the cortex HFC networks showed positive assortativity, while all the other networks showed negative assortativity. However, there are no comparable measurements of assortativity in neural networks based on individual neurons. Given our unique results, it will be worth carrying out comparable measurements in other preparations, including acute slices and in vivo, to further explore these results.

### Wavelet transform and alternative methods

Besides cross-correlation, there are various methods for inferring connectivity based on neuronal activity, including the generalized linear models [69], [70], Granger causality [71]–[74], and transfer entropy and its extensions [25], [75]–[80]. We chose cross-correlation for its simplicity, and we applied wavelet transform to the cross-correlations because we were especially interested in the temporal structure of the neuronal interaction. The wavelet transform is widely used in neuroscience for analyzing continuous signals such as field potentials and for waveform identification in spike sorting [81], [82]. Application of the wavelet analysis to spiking activity is not common, but see [33]. As far as we know, this is the first application of the wavelet transform to cross-correlations of spiking activity. Also, we are currently trying to expand our description of network connectivity to an information theoretic approach by employing transfer entropy [83,84] (Timme et al. 2013, SfN Indianapolis Chapter Meeting, poster presentation).

### Organotypic cultures

The present study was conducted using organotypic cultures [85]. It is known that the gross anatomy of these cultures resembles the general structure found in the original neuronal systems [86]–[88] as well as some detailed structures including neuronal morphology [89], cytoarchitecture [90], [91], and precise intracortical connectivity [92]. There are also similarities to the in-vivo system in physiological aspects: intrinsic physiological properties [93], precisely timed responses [94], UP states [95], oscillations [96], [97], synchrony [96], [98], waves [99], repeating activity patterns [98], [100] and neuronal avalanches [101]. However, there are also several known differences from in-vivo brains such as exuberant innervation of the CA1 region of hippocampus [88], exaggerated excitatory postsynaptic potential (EPSP) [87], aberrant arborization of hippocampal neurons [102], and disrupted layer structure in cortex [103]. Because of these reported differences, we need to cautiously interpret whether or not the results presented here can be carried over to acute slices and in-vivo preparations. However, it should be noted that new biological features such as avalanches were first observed in organotypic cultures and confirmed later in other preparations [101]. It would be interesting to look for the features presented in this paper also in acute slices and in-vivo preparations by using, for example, the computational methods that we developed for the present study.

### The significance of this research in the field

In 2013, a significant US national project in neuroscience called the ‘BRAIN Initiative’ was announced. One of the three goals of this project is “to understand circuit function” [104]. Obviously, this goal requires non-trivial development of data analysis methods and tools. In this paper, we demonstrated that high temporal resolution is an essential key to understand neuronal correlations. Optical methods are indeed useful for recording large numbers of neurons, but their typical temporal resolution for large populations is limited to ∼50 ms. With this temporal resolution, any functional connections above ∼10 Hz (Nyquist frequency of 50 ms resolution) will not be observable as we have seen in Figure 9. Even in the best of circumstances, the temporal resolution of calcium imaging is ∼10 ms (e.g., see Figure 2d and 3f of [105]), with Nyquist frequency ∼50 Hz; it is still out of our putative “synaptic” correlation frequency range (HFC). Electrophysiology can achieve much higher temporal resolution.

We have developed a methodological framework to compare network structure of neuronal networks at multiple frequency ranges. This framework can be applied to any neuronal system as long as recordings of ∼100 neurons with high temporal resolution are possible. Therefore, this same method can be applied to in-vivo and in vitro acute brain slice data as well as to the organotypic cultured brain slice data presented in this paper. It can be applied to different brain regions, with or without genetic modifications and/or pharmacological modifications. Given all these possibilities, this method can serve as a general tool for examining the microscopic functional networks of the brain.
